# An improved and extended dual-index multiplexed 16S rRNA sequencing for the Illumina HiSeq and MiSeq platform

**DOI:** 10.1186/s12863-024-01192-3

**Published:** 2024-01-22

**Authors:** A.K. Larin, K.M. Klimina, V.A. Veselovsky, E.I. Olekhnovich, M.D. Morozov, D.I. Boldyreva, R.A. Yunes, A.I. Manolov, D.E. Fedorov, A.V. Pavlenko, Y.S. Galeeva, E.V. Starikova, E.N. Ilina

**Affiliations:** 1 Lopukhin Federal Research and Clinical Center of Physical-Chemical Medicine of Federal Medical Biological Agency, Moscow, Russia; 2 Research Institute for Systems Biology and Medicine, Moscow, Russia

**Keywords:** 16S rRNA gene sequencing, Microbial community, Indexing, NGS, Batch effects

## Abstract

**Background:**

Recent advancements in next-generation sequencing (NGS) technology have ushered in significant improvements in sequencing speed and data throughput, thereby enabling the simultaneous analysis of a greater number of samples within a single sequencing run. This technology has proven particularly valuable in the context of microbial community profiling, offering a powerful tool for characterizing the microbial composition at the species level within a given sample. This profiling process typically involves the sequencing of 16S ribosomal RNA (rRNA) gene fragments. By scaling up the analysis to accommodate a substantial number of samples, sometimes as many as 2,000, it becomes possible to achieve cost-efficiency and minimize the introduction of potential batch effects. Our study was designed with the primary objective of devising an approach capable of facilitating the comprehensive analysis of 1,711 samples sourced from diverse origins, including oropharyngeal swabs, mouth cavity swabs, dental swabs, and human fecal samples. This analysis was based on data obtained from 16S rRNA metagenomic sequencing conducted on the Illumina MiSeq and HiSeq sequencing platforms.

**Results:**

We have designed a custom set of 10-base pair indices specifically tailored for the preparation of libraries from amplicons derived from the V3-V4 region of the 16S rRNA gene. These indices are instrumental in the analysis of the microbial composition in clinical samples through sequencing on the Illumina MiSeq and HiSeq platforms. The utilization of our custom index set enables the consolidation of a significant number of libraries, enabling the efficient sequencing of these libraries in a single run.

**Conclusions:**

The unique array of 10-base pair indices that we have developed, in conjunction with our sequencing methodology, will prove highly valuable to laboratories engaged in sequencing on Illumina platforms or utilizing Illumina-compatible kits.

**Supplementary Information:**

The online version contains supplementary material available at 10.1186/s12863-024-01192-3.

## Background

Phylogenetic analysis of rRNA nucleotide sequences has emerged as a highly effective approach for investigating microbial communities. In this context, culture-independent profiling predominantly hinges on the sequencing of the 16S rRNA gene, a ubiquitous genetic marker among prokaryotic microorganisms. This culture-independent approach, in contrast to traditional culture-based methods, offers a distinct advantage by enabling the comprehensive detection and analysis of a more extensive array of bacteria and archaea, even within seemingly uncomplicated microbial ecosystems [1].

The prokaryotic 16S rRNA gene, spanning approximately 1500 base pairs, comprises nine variable regions (nucleotides 69–99, 137–242, 433–497, 576–682, 822–879, 986–1043, 1117–1173, 1243–1294, and 1435–1465 for V1–V9, respectively) interspersed between genetically conserved regions. These conserved regions exhibit remarkable similarity across diverse bacterial species, furnishing a dependable foundation for universal amplification primers. Amplification and subsequent sequencing of the variable regions within the 16S rRNA gene facilitate the phylogenetic classification of a broad spectrum of microbial populations [2–4].

The region of the 16S rRNA gene targeted for sequencing on Illumina platforms often depends on the specific research goals and the sample type. However, due to the length limitations of Illumina sequencing reads (= 300 bases), shorter regions of the 16S rRNA gene are typically chosen. This includes single regions like V4 or V6, or combinations like V1–V3 or V3–V5. The V3 and V4 regions are commonly used, either individually or in combination. These regions provide a good balance of variability and length, allowing for effective differentiation between bacterial species while accommodating the shorter read lengths of Illumina sequencing technologies.

The V3-V4 hypervariable regions of the 16S rRNA gene are known for their effective balance in providing taxonomic resolution. A study by Sirichoat et al. indicated that the V3 region showed the greatest richness and diversity in the study of vaginal microbiota, followed by V6-V7 and V4. Studies have shown that while regions like V1-V2 might have a higher resolving power in certain contexts (e.g., respiratory samples), the V3-V4 regions still provide substantial sensitivity and specificity for microbial diversity analysis. Additionally, these regions have shown higher alpha diversity compared to other regions like V7-V9 [5]. Sequencing the V3-V4 regions is cost-effective, especially when using next-generation sequencing (NGS) platforms like Illumina. This economic efficiency makes it a preferred choice for various metagenomic studies. These regions provide a balance between the length of the sequence and the depth of coverage, combining one of the most conserved regions (V4) with one of the most variable regions (V3), which is essential for achieving accurate taxonomic classification [6].

In summary, the V3-V4 region of the 16S rRNA gene is advantageous for sequencing on platforms like Illumina due to its effective balance of taxonomic resolution and diversity analysis, suitability for high-throughput sequencing methods, cost-efficiency, comparative advantages over other hypervariable regions, and broad applicability across different ecological studies [7].

To target this region, researchers commonly employ the 341F–805R primer set, generating amplicons approximately 465 base pairs in length. The Illumina MiSeq platform, offering the capacity to sequence up to 600 nucleotides from both ends of an amplicon [(300 bp)×2], conveniently accommodates such amplicons with full coverage.

However, limitations arise when considering the Illumina HiSeq platform. The use of the Illumina Rapid SBS kit restricts sequencing to a maximum of 500 cycles per run. Furthermore, the commercial indices available for use permit the pooling of no more than 384 samples. These constraints collectively impede the sequencing of sample sets exceeding 384 in a single run. Consequently, researchers resort to sequencing samples in a series of independent runs. This practice, unfortunately, paves the way for the accumulation of batch effects, which are systematic errors arising during sequencing and capable of inducing spurious correlations between biological samples. These batch effects primarily stem from variations in sample preparation, reagent batches, and sequencing runs, all of which contribute to the emergence of these unwanted artifacts [8].

In an endeavor to mitigate the impact of batch effects, our study has undertaken a pioneering approach. We have synthesized a set of 10-base pair dual indices and developed a sequencing method capable of spanning 600 cycles on the HiSeq platform. This innovative methodology allows for the continuous analysis of the V3-V4 regions of the 16S rRNA gene within 1,711 samples collected from human oropharyngeal, mouth cavity, dental, and fecal sources. These samples are sequenced on the Illumina HiSeq 2500 platform, marking a substantial step toward minimizing the influence of batch effects on our results.

## Results

### Indices

The distinctive 10-base pair indices, subject to error correction and detection capabilities, were created using the R language function ‘create.dnabarcodes’ from the DNABarcodes package [9]. The creation of these indices adhered to the following parameters: ‘n’ set to 10 (representing the desired length in base pairs), ‘dist’ defined as 6 (ensuring a minimal distance between the indices), and the employment of the ‘heuristic’ option with the ‘ashlock’ setting (utilizing an evolutionary heuristic algorithm to generate the sequence set). Subsequently, 88 sequences were randomly selected using the ‘sample’ function (for more details, refer to Supplementary Table S1).

### Validation of samples

In the initial phase, we sequenced the prepared 1,711 16S libraries on the Illumina MiSeq instrument (Illumina, USA) to assess the distribution of reads per sample. The number of reads per sample exhibited a range spanning from 104 to 31,592, with a mean value of 4,003 reads per sample. This mean value, however, may pose limitations for the comprehensive reconstruction of complex and diverse microbial communities. To evaluate the effect of index-hopping on our results, we employed the *Escherichia coli* strain as a positive control. In total, 48,445 reads were obtained for this control sample, and only 94 reads (constituting 0.00194% of the total) did not belong to the *E. coli* amplicon sequence variant (ASV). This indicates a potential index hopping rate of 0.2%, which is essentially negligible.

**Fig. 1:**
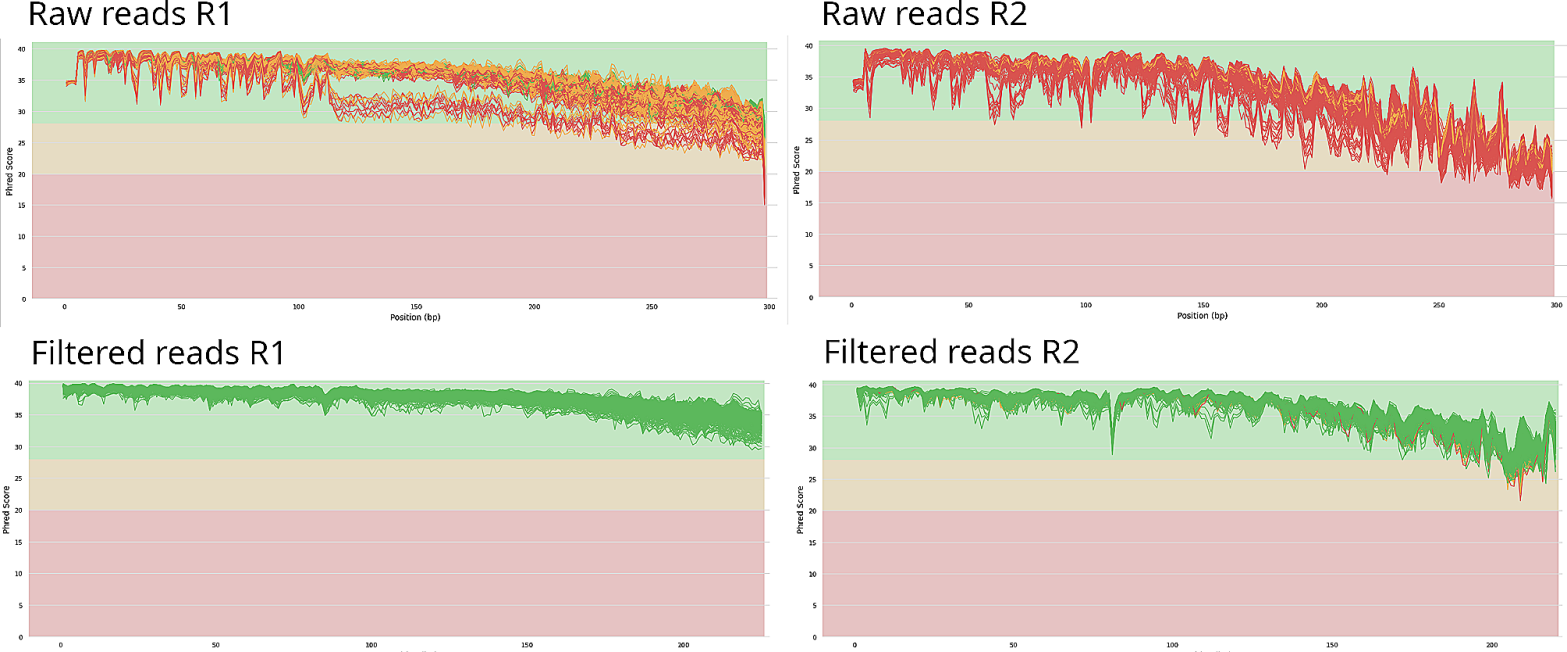
Quality profiles of raw reads and filtered reads obtained on HiSeq instrument

Subsequently, the same set of samples was processed on the HiSeq instrument. In total, we generated 125,392,900 reads with an average length of 600 base pairs, resulting in an average of 73,286 reads per sample. Following quality filtering, approximately 16% of the raw reads were removed. Of the remaining paired reads, 93% were successfully merged, with an additional 8% eliminated as chimeras. The final dataset comprised 89,758,460 reads, equivalent to 72% of the raw reads, and resulted in an average of 52,459 reads per sample (± 26,104, as illustrated in Fig. 1).

**Fig. 2:**
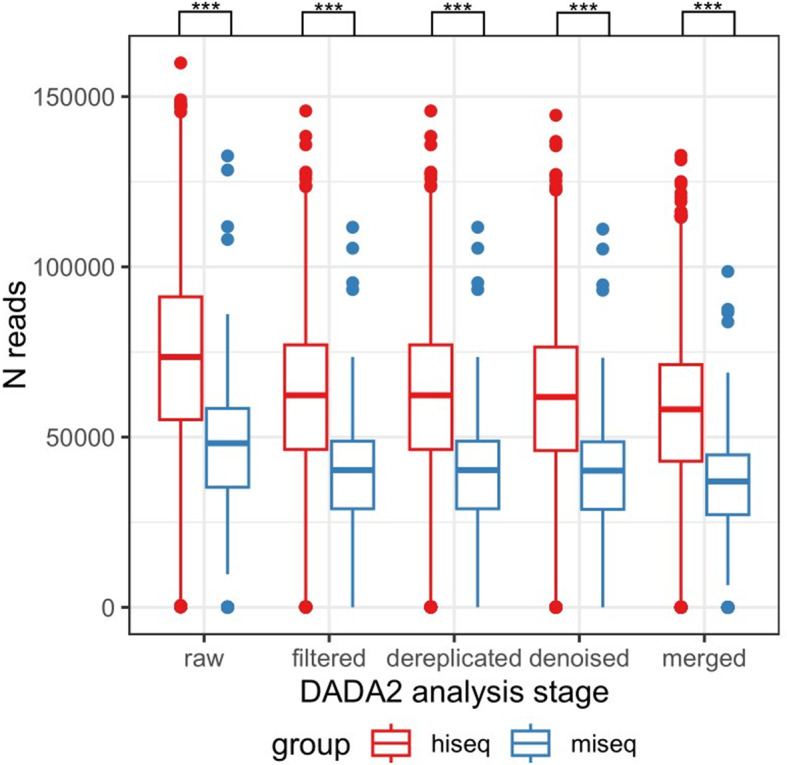
Read counts at various stages of the DADA2 pipeline for samples sequenced on both the HiSeq and MiSeq platforms. The data processing was conducted using the DADA2 pipeline

The read counts at each stage of the DADA2 pipeline revealed notable disparities, as depicted in Fig. 2. It is evident that the depth and quality of the sequencing play pivotal roles in dictating the quantity of reads subjected to removal during the quality filtering phase. Significantly, owing to the substantially higher throughput of the HiSeq platform in comparison to the MiSeq, the HiSeq data exhibited a more pronounced count of reads eliminated during quality filtering than its MiSeq counterpart.

Furthermore, it is pertinent to highlight that the samples sequenced on the HiSeq platform demonstrated considerably superior mean quality scores for both the forward and reverse reads.

Dereplication, the process of reducing identical sequences to a single representative sequence, plays a crucial role in data analysis. The number of unique sequences remaining after dereplication is influenced by the depth of sequencing and the complexity of the bacterial community. A comparative analysis of the two datasets following dereplication revealed a noteworthy distinction: the HiSeq data exhibited a higher count of unique sequences in contrast to the MiSeq data, as depicted in Fig. 2.

**Fig. 3:**
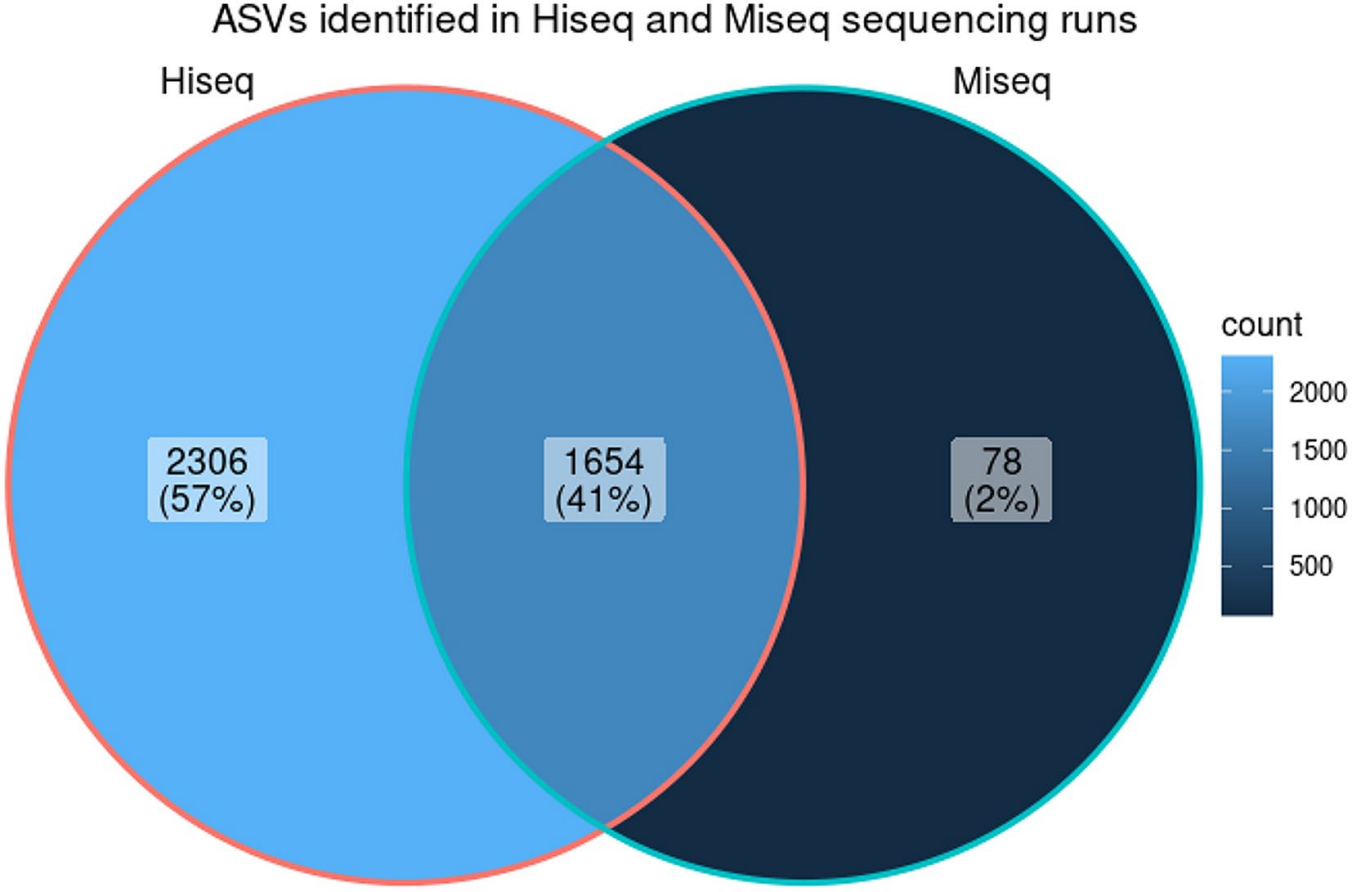
Amplicon sequence variants identified in HiSeq and MiSeq runs

In the HiSeq dataset, we identified 3,960 unique Amplicon Sequence Variants (ASVs) present in at least 1% of the samples, in contrast to the MiSeq dataset, which contained 1,732 ASVs meeting this criterion. A mere 78 sequences were unique to the MiSeq run, whereas the HiSeq run yielded 2,306 unique sequences. Interestingly, 1,654 sequences were common to both datasets, as illustrated in Fig. 3.

## Discussion

A 10-nucleotide index size was chosen as a judicious compromise, balancing the need for sufficient length to avoid misinterpretation of the index due to random errors while preserving the informative portion of the sequence. The selection of 10-base indices offers a significantly larger pool of unique index combinations compared to alternative index systems, such as 6-base or 8-base indices. This becomes especially pivotal in large-scale studies, where a multitude of samples must be processed. Utilizing 10-base indices diminishes the risk of index collisions, thus ensuring accurate sample identification.

In terms of read length, the MiSeq platform held an advantage over the HiSeq platform due to its capacity to produce up to 300 bp reads, compared to the 250 bp reads achievable with the HiSeq platform [10]. Furthermore, the HiSeq platform excelled by generating up to 1 billion reads, contrasting with the 25 million reads attainable with the MiSeq platform. Increased throughput translates to augmented sequencing coverage and heightened statistical power for the identification of variations within bacterial communities, providing another edge to the HiSeq platform [11].

Additionally, the HiSeq platform consistently delivered a higher average number of quality-filtered sequences per sample. This increased data yield and enhanced sequencing depth translate to a greater capacity for sequencing multiple samples in a single run, further solidifying the HiSeq’s suitability for large-scale experiments. In contrast, the MiSeq platform is better suited for smaller-scale experiments necessitating swift turnaround times.

It’s worth noting that 16S rRNA sequencing results may vary across experiments due to the ‘batch effect’, a potential source of bias introduced at various stages of the experimental workflow, including DNA extraction, amplification, and sequencing. Sequencing a larger number of 16S rRNA samples often presents challenges, as it necessitates conducting a series of independent runs, elevating the risk of batch effects that can lead to inconsistent results and skewed data. Importantly, in addition to biases introduced during DNA extraction and library preparation, batch effects can also emerge during the sequencing process due to variations in instrument performance, reagent lots, and environmental conditions.

To address and mitigate these issues arising from batch effects, computational methods have been developed. These methods are designed to identify and rectify batch effects while preserving the underlying biological variability in the data. One widely adopted approach for batch effect correction is the ComBat algorithm, initially developed for microarray data analysis [12]. Over time, this algorithm has been adapted for 16S rRNA sequencing studies, where it has demonstrated efficacy in addressing and correcting batch effects [13].

In the realm of 16S rRNA sequencing studies, another computational method employed for batch effect correction is RUVSeq, which expands to ‘remove unwanted variation using negative controls’. RUVSeq leverages a set of negative control taxa, which are not expected to exhibit differential expression, to make adjustments for batch effects [14, 15]. This approach has proven effective in the context of microbiome data; however, it necessitates the presence of a predefined set of negative control taxa, either as spike-ins or as empirical negative control taxa [16].

To mitigate the impact of batch effects in 16S rRNA sequencing studies, several strategies are available. One tactic involves incorporating replicate samples or technical controls in each sequencing run, facilitating an assessment of data variability and reproducibility. Another strategy revolves around normalization methods designed to counteract batch effects by scaling the data based on the distribution of control samples.

It is essential to acknowledge that while multiple strategies and computational methods can help alleviate batch effects, complete elimination remains challenging. The effectiveness of these methods may vary depending on the specifics of a 16S rRNA sequencing study and its design. Thoughtful experimental design, including the inclusion of cross-batch controls, can significantly reduce the influence of batch effects and simplify their correction.

Furthermore, it is crucial to exercise caution when relying solely on computational methods, as they may instill unwarranted confidence in downstream analyses. One surefire way to circumvent batch effects is to sequence all samples in a single sequencing run, obviating the need for correction methods.

For this purpose, we have devised a comprehensive list of 10-base indices. This approach empowers us to sequence and analyze a large number of samples within a single run. The utilization of these 10-base dual indices has demonstrated its effectiveness in ensuring accurate sample identification and minimizing the occurrence of false positives.

## Conclusions

Amplicon sequencing continues to be a widely embraced method for the examination of microbial communities in both clinical and scientific domains. Its cost-effectiveness renders amplicon sequencing conducive for large-scale comparative analysis across numerous samples. We firmly believe that our custom list of 10-base pair indices, alongside our adapted demultiplexing protocol, holds substantial value for laboratories utilizing Illumina sequencing instruments such as NextSeq, HiSeq, MiSeq, and others. Furthermore, our approach is adaptable to any DNA sequencing platform compatible with Illumina technology, extending its utility to platforms like GenoLab M from GeneMind Biosciences Company.

As evident from the results presented in Fig. 2, the read counts at various stages of the DADA2 pipeline exhibit marked disparities. This discrepancy is heavily influenced by both the sequencing depth and quality. Notably, due to the higher throughput typically associated with HiSeq sequencing, a larger number of reads are subjected to removal during quality filtering, compared to MiSeq data. Moreover, it is worth highlighting that data derived from samples sequenced on the HiSeq platform consistently display significantly higher mean quality scores for both forward and reverse reads.

## Methods

### Oligos

We employed the DNABarcodes package [9] to create a distinctive set of 10-nucleotide indices (please refer to Supplementary Table S1 for details). The oligos designed for amplification were synthesized by Evrogen, a reputable company in this domain (evrogen.com).

### Samples

We utilized genomic DNA sourced from 1,711 distinct clinical specimens, categorized as follows: 205 nasopharyngeal swabs, 474 mouth swabs, 359 mouth washes, 407 dental plaques, and 265 fecal samples. These DNA samples were generously provided by Moscow State University of Medicine and Dentistry (MSUMD).

DNA was extracted by employing the MagMAX™ DNA Multi-Sample Ultra 2.0 Kit (Thermo Fisher Scientific, USA) in conjunction with the King Fisher Flex Purification System (Thermo Fisher Scientific, USA), according to the manufacturer’s protocol. Subsequently, the quantification of DNA was carried out using the Qubit 4 fluorometer, utilizing the Quant-iT dsDNA BR Assay Kit (Thermo Fisher Scientific, USA).

### Library preparation

In this protocol, gene-specific sequences targeting the 16S V3 and V4 region were selected from the publication by Klindworth et al. [17]. The 16S library preparation and sequencing adhered to the Illumina protocol [18].

For the amplification of the extracted DNA (ranging from 1 to 5 ng), standard 16S rRNA gene primers, designed to complement the V3-V4 region and incorporating 5’-Illumina adapter sequences (16S Amplicon PCR Forward Primer = 5’ TCGTCGGCAGCGTCAGATGTGTATAAGAGACAGCCTACGGGNGGCWGCAG and 16S Amplicon PCR Reverse Primer = 5’ GTCTCGTGGGCTCGGAGATGTGTATAAGAGACAGGACTACHVGGGTATCTAATCC), were employed. These primers were sourced from Evrogen, Russia. The amplification was conducted using the Tersus Plus PCR kit (Evrogen, Russia) in a total volume of 25 µl.

The first amplification step involved the following PCR conditions: an initial denaturation at 95°C for 2 min, followed by 27 cycles of denaturation at 95°C for 30 s, annealing at 60°C for 30 s, extension at 72°C for 30 s, and concluded with a final extension at 72°C for 2 min. Subsequently, the reaction was cooled to 4°C.

The second amplification step, responsible for attaching dual indices and Illumina sequencing adapters, followed a similar protocol. It initiated with an initial denaturation at 95°C for 2 min and proceeded with 8 cycles, which involved denaturation at 95°C for 30 s, annealing at 60°C for 30 s, extension at 72°C for 30 s, and a final extension at 72°C for 2 min, ending with cooling to 4°C.

Subsequently, individual amplicons underwent PCR indexing and were subsequently pooled. The size distribution and quality of the libraries were assessed utilizing a high-sensitivity DNA chip (Agilent Technologies), and the libraries were quantified using the Quant-iT DNA Assay Kit, High Sensitivity (Thermo Scientific, USA).

### Sequencing on illumina platforms

The DNA libraries underwent sequencing using the MiSeq instrument (Illumina, USA) with the MiSeq reagent kit v3 (Illumina, USA).

Sequencing of the DNA libraries was also conducted on the HiSeq 2500 platform (Illumina, USA), in accordance with the manufacturer’s recommendations. For this purpose, we employed the following reagent kits: HiSeq Rapid PE Cluster Kit v2, HiSeq Rapid SBS Kit v2 (500 cycles), HiSeq Rapid SBS Kit v2 (200 cycles), and HiSeq Rapid PE FlowCell v2. Additionally, a 2% PhiX spike-in control was included in the process.

As we were sequencing a 16S rRNA library, necessitating 600 cycles (300 + 300 paired-end reads), we followed Illumina’s guidelines. The sequencing run was initiated by configuring the run recipe as PE 300 on the HiSeq 2500, using the HiSeq Rapid PE FlowCell v2, HiSeq Rapid PE Cluster Kit v2, and HiSeq Rapid SBS Kit v2 (500 cycles). Upon depletion of the 500-cycle reagent kit, the HiSeq Control Software automatically paused the run. Subsequently, we replaced the empty cartridge with reagents from the HiSeq Rapid SBS Kit v2 (200 cycles), following the software instructions for the HiSeq 2500, and then resumed the run.

### Primary analysis

We processed the sequencing data using several bioinformatics tools and packages. Firstly, Illumina adapters were removed from the reads using Trimmomatic [19], and the primers were eliminated using cutadapt [20]. Subsequently, we filtered the reads employing the filterAndTrim function from the DADA2 package [21] with the following parameter settings: truncLen_f: 225, truncLen_r: 220, trimLeft_l: 0, trimLeft_r: 0, maxN: 0, maxEE_f: 2, maxEE_r: 2, truncQ: 2, and rm_phix: ‘TRUE’.

ASV (Amplicon Sequence Variant) inference was carried out utilizing DADA2, and the read pairs were merged with a minimum overlap of 18 base pairs. Lastly, chimeric sequences were removed through the removeBimeraDenovo function, employing the consensus method.

### Electronic supplementary material

Below is the link to the electronic supplementary material.

Supplementary Material 1: Table S1. 88 custom 10-nucleotide indexes generated using the DNABarcodes package

## Data Availability

Table S1 containing the designed oligonucleotide indices is provided in the Supplementary Data. Additionally, the datasets generated and analyzed during this study have been made accessible in the ‘MGMSU Covid 16s’ repository at https://www.ncbi.nlm.nih.gov/bioproject/PRJNA989180 with Accession ID: 989180.
