# Supplementary material for: An improved and extended dual-index multiplexed 16S rRNA sequencing for the Illumina HiSeq and MiSeq platform
Source: BMC Genom Data. 2024 Jan 22;25:8. doi: 10.1186/s12863-024-01192-3 (PMC10804484; doi:10.1186/s12863-024-01192-3)
Supplement: Supplementary file 1 — Supplementary Material 1: Table S1. 88 custom 10-nucleotide indexes generated using the DNABarcodes package [file 12863_2024_1192_MOESM1_ESM.docx]

Table S1. 88 custom 10-nucleotide indexes generated using the DNABarcodes package

| **Id** | **Sequence** |
| --- | --- |
| 1 | AGGACCGGTT |
| 2 | TGCGTACCTG |
| 3 | GGTGTTGGAA |
| 4 | CCAGACGCAA |
| 5 | GTGAGTTAAC |
| 6 | ATACACTACC |
| 7 | CTTCGGACAA |
| 8 | ATCGTCCGCA |
| 9 | GACTATTGCT |
| 10 | GGACAGCTAA |
| 11 | TTGCTGTCCT |
| 12 | AACCGCGAAG |
| 13 | ATATGTCGAG |
| 14 | GCTCACTGGA |
| 15 | AGCAGCTTGC |
| 16 | ACTCCTAGTC |
| 17 | CTAACTGGCA |
| 18 | TATCCGTTAC |
| 19 | ATAATGAGGC |
| 20 | TGAACAGAAG |
| 21 | CAGAGCAAGA |
| 22 | GATCGAATGG |
| 23 | GAATTCGACA |
| 24 | GTTAGCGTTA |
| 25 | TACAGTGGTC |
| 26 | AATACACGGA |
| 27 | GTCTTCTTAG |
| 28 | CACGGACTAA |
| 29 | AGACTTGTCG |
| 30 | TCCACCTACA |
| 31 | TTCGGTAACG |
| 32 | GCAAGAAGAA |
| 33 | CTGGTGGAAG |
| 34 | GTCTAGAAGA |
| 35 | AGAGCTAAGA |
| 36 | TGGTTGCGGA |
| 37 | CTTACGTAGT |
| 38 | CACATAAGCG |
| 39 | TCGCTCATAA |
| 40 | AAGGCAGACC |
| 41 | CTCGACATTC |
| 42 | GTCACACTCC |
| 43 | AATTGGTCCG |
| 44 | CGTTAACGTT |
| 45 | CTGTCAACGG |
| 46 | ATGCCGGTGA |
| 47 | TCTGGAGAGA |
| 48 | GAAGCCAGTG |
| 49 | GACACGGCAA |
| 50 | GCCATTGAGG |
| 51 | GCATCATAGC |
| 52 | CCGTAATTCA |
| 53 | ACATACATGG |
| 54 | CAGCTTCGAC |
| 55 | CGAAGGTGTG |
| 56 | AGTAAGGACA |
| 57 | TGAGATCGCC |
| 58 | CGCTCCAGAA |
| 59 | GAGGTATCGA |
| 60 | TCACGACTTC |
| 61 | CTTGGATGCC |
| 62 | GGCGAAGTGT |
| 63 | GCTCCACCAT |
| 64 | ACGCGTCACA |
| 65 | CCACAGAGCT |
| 66 | CGAGCCTTCT |
| 67 | TTATGCTCGA |
| 68 | CGCTTGTACC |
| 69 | CCTTCCGATG |
| 70 | AGTATCACAG |
| 71 | AAGGAGAGAA |
| 72 | GTGTTAGGTC |
| 73 | CGTCATTAAG |
| 74 | TGGCAGAATC |
| 75 | TCTAAGCGAG |
| 76 | ATTGATGCGG |
| 77 | AACCTTACGT |
| 78 | TTCCAAGGAA |
| 79 | TGTTCTGCCT |
| 80 | TTGGCTCCAA |
| 81 | GCGGTCCATT |
| 82 | TAGAACGCCG |
| 83 | ACCAGGAATT |
| 84 | TAACCAACCA |
| 85 | CGCAATCCGA |
| 86 | TGTTGCCAAC |
| 87 | GATGAGCCTC |
| 88 | TGGAGAATCT |
